# Paleoecology of extinct species

**DOI:** 10.1186/s12862-023-02170-6

**Published:** 2023-10-06

**Authors:** Michael Pittman, Yucheng Wang

**Affiliations:** 1 School of Life Sciences, The Chinese University of Hong Kong, Shatin, Hong Kong SAR China; 2 Department of Zoology, University of Cambridge, Cambridge, UK; 3 Group of Alpine Paleoecology and Human Adaptation (ALPHA), State Key Laboratory of Tibetan Plateau Earth System, Resources and Environment (TPESRE), Institute of Tibetan Plateau Research, Chinese Academy of Sciences, Beijing, China; 4 Lundbeck Foundation GeoGenetics Centre, Globe Institute, University of Copenhagen, Copenhagen, Denmark

## Abstract

Recent developments, including new imaging and ancient environmental DNA (aeDNA) technologies, are providing unprecedented insights into the past, which can also help researchers predict future ecological change. BMC Ecology and Evolution has launched a new article Collection on the “Paleoecology of extinct species” to provide an open-access resource for all interested in this multidisciplinary field.

## Main text

Paleoecological studies offer captivating glimpses into long-lost worlds and uncover drivers underlying long-term biodiversity patterns, allowing researchers to predict future ecological change and inform conservation decisions. Researchers can look more clearly and deeper into the past with recent technological and methodological advances, including new imaging and ancient DNA techniques. *BMC Ecology and Evolution* has launched this new article Collection on the “Paleoecology of extinct species” to highlight advancements and efforts to tackle challenges in this multidisciplinary field.

The accuracy of paleoecological reconstructions can be improved with further innovation, cross-pollination with modern ecology, and data integration. A prime example is using Laser-Stimulated Fluorescence on fossils inspired by confocal microscopy. This has revealed otherwise hidden soft tissues, providing key new insights beyond existing evidence [1], especially in early flight studies [2]. Another example is dental microwear analysis to study extinct species, which has improved with methodological developments and data from modern animals. This has provided clearer dietary insights where they were previously ambiguous or speculative [3]. Recent efforts promoting quantitative, multi-evidence approaches for reconstructing dietary ecology have helped to reemphasise the benefits of data integration, including dietary insights that would not have been possible from single lines of evidence alone. Improved data integration also allows better identification of remaining knowledge gaps [4]. For example, researchers have better reconstructed early fossil birds’ diets by integrating body mass data, claw morphometrics, jaw mechanical advantage, and jaw strength (via finite element analysis) [5]. We seek studies that build on these outlined aspects of innovation, cross-pollination and data integration to uncover important new ecological insights. Studies describing exceptionally preserved specimens that fill longstanding knowledge gaps are also encouraged as they underscore the continued importance of new fossil discoveries.

Closer to the present, ancient DNA preserved in all kinds of sediments, namely the ancient environmental DNA (eDNA) or sedimentary ancient DNA (sedaDNA), offer the chance to sample and sequence degraded nucleic acids of some extinct species. Advances in the recovery and analysis of biological sequence data from these degraded nucleic acids have opened considerable new avenues of research in molecular palaeoecology and evolution. Ancient eDNA methods can now be applied to study past biodiversity at an ecosystem scale [6, 7]; to zoom in on nuclear genomes of extinct fauna [8]; and reach deeper in the past than any other paleo-genomic study [9]. In particular, this has provided considerable scope to re-evaluate the spatial and temporal distribution of extinct taxa independent of the preservation of macro-remains, notably Pleistocene megafauna such as mammoths [7, 10]. Consequently, ancient eDNA has morphed into a highly dynamic and rapidly growing topic of palaeoecological research.

Nonetheless, significant conceptual and technical challenges remain in ancient eDNA research. Such challenges include avoiding contamination with modern DNA, excluding the effects of redeposition and leaching of DNA molecules across sediment stratums, PCR bias, incomplete reference databases, difficulties in discriminating phylogenetically close species (e.g., rodent taxa), lack of proper and efficient methods for ancient metagenome analysis (i.e., an averaged genome of multiple individuals of a species), and accurate taxonomic assignment of reads, particularly from degraded and short sequences. Furthermore, there is a growing need for computationally efficient yet accurate means of processing the exponentially increasing volume of data produced by modern reference genome sequencing. We encourage the submission of articles that deepen and broaden molecular palaeoecological research and make headway in addressing the major conceptual and technical challenges outlined.

BMC Ecology and Evolution welcomes submissions to a new article Collection on the ‘Paleoecology of extinct species’. The Collection aims to bring together paleoecology research from around the world, highlighting advancements and efforts to tackle the numerous challenges faced by researchers working in the field.

## Data Availability

Not applicable.
